# Unexpected Liver Embryonal Sarcoma in the Adult: Diagnosis and Treatment

**DOI:** 10.1155/2018/8362012

**Published:** 2018-06-04

**Authors:** Maurizio Pinamonti, Federico Vittone, Francesco Ghiglione, Andrea Borasi, Stefano Silvestri, Sergio Coverlizza

**Affiliations:** ^1^Department of Pathology, Humanitas Gradenigo Hospital, Turin, Italy; ^2^Department of Pathology, Civil Hospital of Ivrea, Ivrea, Italy; ^3^Department of Surgery, Humanitas Gradenigo Hospital, Turin, Italy; ^4^Department of Surgery, Santo Spirito Hospital, Casale Monferrato, Italy

## Abstract

Undifferentiated embryonal sarcoma of the liver is a malignancy with poor prognosis observed more frequently in children between 6 and 10 years old and very rarely found in adults. We present a case of embryonal sarcoma of the liver in a 60-year-old woman without significant medical history who presented to our attention with constitutional symptoms. Preoperative assessments did not show alterations in blood chemistry or tumor markers. Imaging studies showed a huge mass lying in the right abdominal quadrants, strictly adherent to the liver. The tumor was partially cystic with a thickened wall, sporadic contrast enhancement, and solid component. The patient underwent excision of the mass with associated liver bisegmentectomy S5-S6. Postoperative course was uneventful. The definitive histological diagnosis revealed the presence of embryonal sarcoma of the liver. We describe the clinical, histopathological, and therapeutic options adopted in the multimodal treatment of this disease.

## 1. Introduction

Undifferentiated embryonal sarcoma of the liver (UESL) is an extremely rare neoplasm in adults with less than 60 cases described so far in the literature [1–7]. It is most typically found in children between 6 and 10 years of age, where it represents the third most common primary liver malignancy after hepatoblastoma and hepatocellular carcinoma (HCC) [8, 9]. UESL is an aggressive tumor with a tendency for local infiltration and systemic metastases. Although, like many other mainly pediatric neoplasms, it is potentially treatable, and prognosis is good if the lesion is diagnosed at an early stage [10]. Unfortunately, the clinical and radiological manifestations are nonspecific, and the lesion might be misrecognized, leading to a delay in the appropriate therapy [5]. Surgery is the first treatment option, and radical resection should be considered in all cases [2].

We describe a case of a middle-aged woman with abdominal mass in the absence of specific symptomatology submitted to laparotomy with a final diagnosis of embryonal sarcoma of the liver.

## 2. Case Report

**Figure 1:**
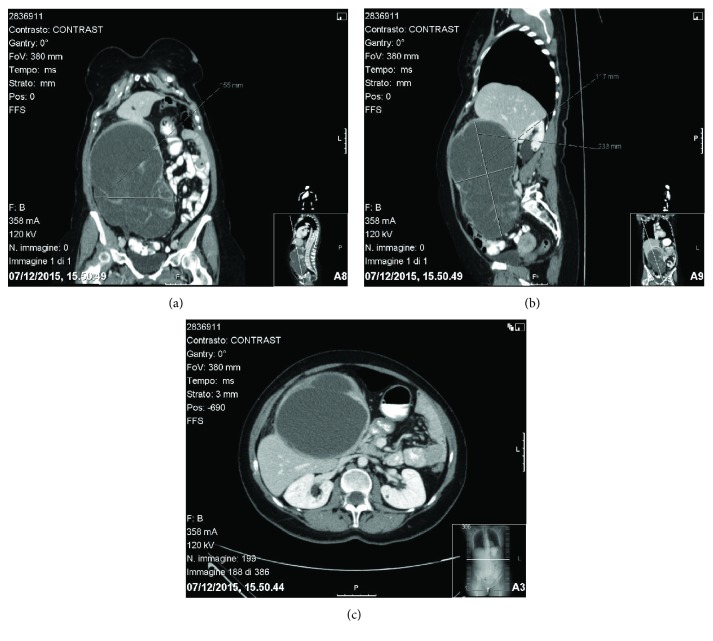
(a, b, c) A huge, partially cystic mass with heterogeneous contrast enhancement is seen in the right abdominal quadrant, adjacent to the liver (CT scan).

A 60-year-old woman came to our attention for abdominal pain, distension, and weight loss (about 6 kg in two months). Abdominal examination revealed a bulky mass occupying the right abdominal quadrants. She was submitted to laboratory routine tests without evidence of chronic liver disease and no alterations of serum tumor markers. Computed tomography (CT) scan confirmed the presence of an expansive mass (15 × 12 × 23 cm), poorly separable from the surrounding liver parenchyma, with heterogeneous contrast enhancement. The lesion was partially cystic with thickened walls and an intralesional solid component (Figure 1).

After multidisciplinary discussion with radiologist and oncologist, indication to surgery was given and the patient underwent laparotomy. At exploration, a well-defined, partially cystic tumor was found originating from the fifth and sixth hepatic segments and adherent to—but not infiltrating—the right colon and omentum. En bloc resection of the mass and a S5-S6 liver bisegmentectomy were performed. The postoperative period was uneventful, and the patient was discharged on sixth postoperative day.

**Figure 2:**
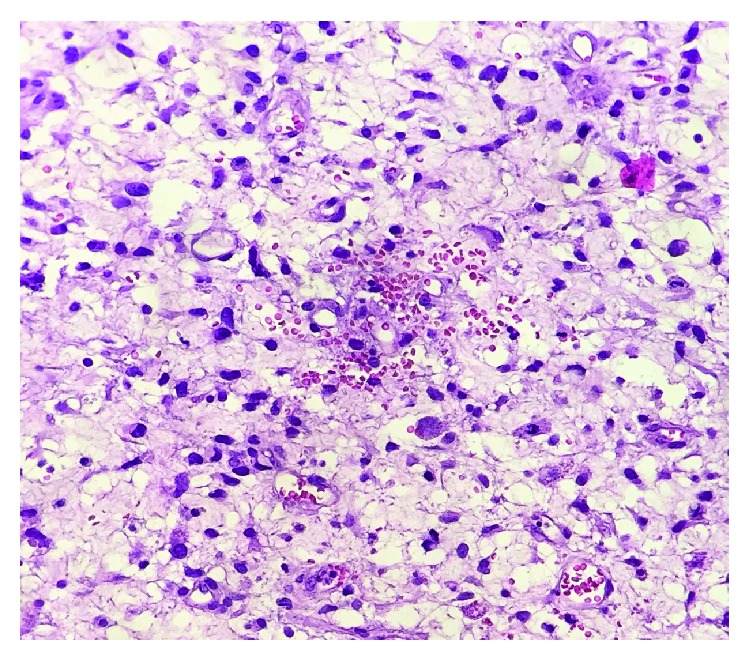
Mixture of highly atypical spindle and giant cells. The larger cells often contain numerous intracytoplasmic hyaline globules (hematoxylin and eosin, 20x magnification).

**Figure 3:**
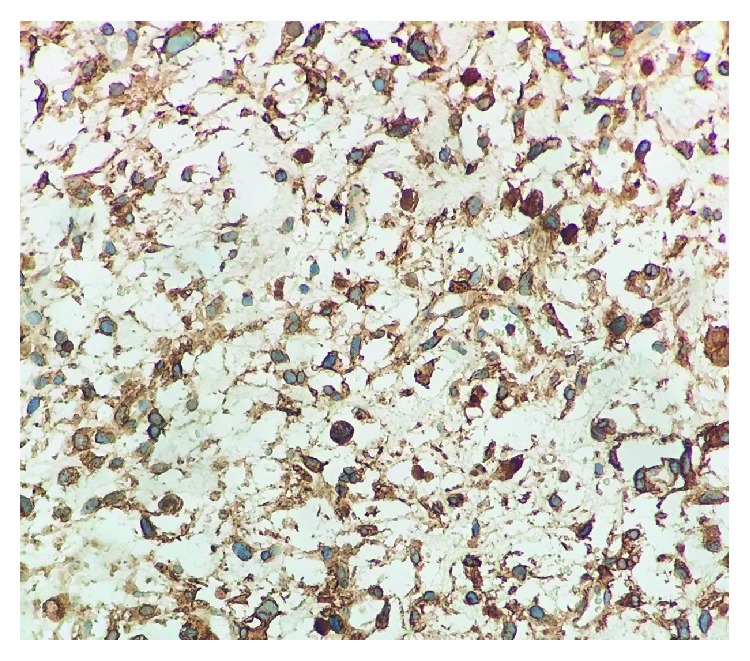
Tumor cells show strong membranous expression of CD10 (CD10 immunohistochemistry, 20x magnification).

The surgical specimen consisted of a lobulated yellow-reddish neoplasm sized 33 × 19 × 11 cm, with gelatinous cystic and hemorrhagic areas on cut surface. A fibrous discontinuous pseudocapsule separated the tumor from the adjacent compressed liver parenchyma. Microscopically, the tumor was composed of stellate or spindle shaped cells with bizarre morphology and ill-defined outlines, loosely arranged in an abundant myxoid matrix (Figure 2). Scattered tumor cells with marked nuclear abnormalities and hyperchromasia, as well as multinucleated giant cells, were present. Atypical mitotic figures were easily found. Characteristically, tumor cells showed multiple, different-sized, eosinophilic, PAS-positive globules in the cytoplasm. Immunostainings revealed CD10, CD68, and vimentin expression in tumor cells, with focal and weak expression of wide spectrum cytokeratins (AE1/AE3) (Figure 3). These features were sufficient for the diagnosis of UESL.

After the definitive histological diagnosis and multidisciplinary evaluation, the oncologist proposed an adjuvant therapy of six cycles with vincristine, actinomycin D, and cyclophosphamide, which was accepted by the patient. 30 months after surgery, she is alive without signs of recurrence.

## 3. Discussion

Stocker and Ishak first introduced the term undifferentiated embryonal sarcoma in 1978 to describe a mesenchymal hepatic tumor without any sign of specific differentiation [8]. In the past 50 years, less than 60 adult cases have been reported [1–7], with a mean age of 25 and the oldest patient reported aged 84 [4]. A slight female predilection has been reported in the adult, differently to what observed in the childhood.

Already in its first description, a mesenchymal origin was proposed and a possible precursor lesion was identified in mesenchymal hamartoma (MH) of the liver [8]. Supporting this theory, similar genetic abnormalities were described in both MH and UESL and especially chromosome 19 aberrations [11–13]. TP53 mutations were identified in UESL, suggesting a role in the malignant transformation of the lesion [3].

UESL may occur in any part of the liver but is most commonly found in the right lobe as a single, rapidly growing mass. Symptoms are directly correlated with the neoplasm's growth and include pain, abdominal swelling, weight loss, fever, anorexia, vomiting, diarrhea, lethargy, constipation, and respiratory distress [8, 10, 14]. There is no any specific marker in the serum, although some authors have registered an increase of hepatic enzymes and cancer antigen 125 in few cases [2].

Radiological imaging is nonspecific but is generally able to detect a nodular mass in the liver. Ultrasonography usually shows a large mass with solid and cystic components which could be mistaken for an abscess or an echinococcal cyst. CT scan reveals an inhomogeneous mass with a hypodense component and eccentric abnormal contrast intake. Magnetic resonance imaging usually confirms the CT findings but is more useful for the identification of vascular invasion, biliary obstruction, and hilar adenopathy [14]. Precise localization of the tumor and its relationship with the major vessels are crucial in defining its resectability, which represents the most important prognostic factor in patients with UESL.

The macroscopic aspect of UESL is a nodular mass, which is usually solitary but may seldom be multiple and appears to be well demarcated with a fibrous pseudocapsule deriving from the compressed hepatic parenchyma around the lesion. On the cut surface, the neoplasm shows solid gray-white areas intermingled with gelatinous cystic spaces. Yellowish, poorly demarcated, necrotic areas as well as red-brown hemorrhagic areas are common. Microscopically, the neoplasm is composed of a population of round, spindle, or stellate cells with inconspicuous nucleoli and indistinct cellular borders in a myxoid matrix. Scattered cells with hyperchromatic and bizarre nuclei as well as multinucleated giant cells may be present. The mitotic index is typically high, and atypical mitoses are frequently observed. A peculiar morphological feature is represented by variable-sized eosinophilic globular inclusions in the cytoplasm and in the extracellular matrix. These are PAS-positive and diastase resistant [8, 15, 16].

Immunohistochemistry is generally required in order to reach the definitive diagnosis and rule out other possible mimickers. A specific immunophenotype has still not be identified in UESL. Tumor cells usually express vimentin, desmin, CD68, B-cell lymphoma 2 (BCL2), *α*1-antitripsine, and CD10, while negative for myogenin, CD34, CD117 (C-kit), hepatocyte paraffin 1 (Hep-par1), anaplastic lymphoma kinase 1 (ALK1), and S-100 [17]. Some authors report a focal expression of cytokeratins [18] and also positivity for Glypican 3 [19], which is a diagnostic marker expressed by hepatoblastoma and HCC. No one marker is diagnostic alone, and the use of a panel, including at least two or three antibodies commonly expressed by UESL, together with those needed for the exclusion of other entities, is strongly recommended [16].

Differential diagnoses should be weighted considering the age of the patient, since each hepatic lesion presents more frequently in a certain age group. Lesions to be carefully excluded in children include hepatoblastoma, mesenchymal hamartoma, and embryonal rhabdomyosarcoma of the biliary tree. In the adult, the main differential diagnoses are with gastrointestinal stromal tumor (GIST), HCC with sarcomatoid aspects, and other high-grade sarcomas, especially leiomyosarcoma and angiosarcoma. GIST might be similar in the morphology of tumor cells, but classically express CD34 and C-kit in tumor cells [20]. Distinction from HCC is generally based on morphology, for the presence of intracellular bile deposits and nests of polygonal hepatocytes, but immunohistochemistry could be necessary when these features are not evident. Leiomyosarcoma could enter the differential diagnosis for the presence of focal storiform growth pattern and for the common expression of myogenic differentiation markers, such as vimentin, desmin, smooth muscle actin, and muscle-specific actin. Nevertheless, UESL is typically negative for myogenin and h-caldesmon, which allow a differential diagnosis with a leiomyosarcoma [1, 20]. Angiosarcoma of the liver typically expresses markers of vascular differentiation (CD31, CD34, and coagulation factor VIII), which are negative in most UESL [20]. Liver metastasis of malignant melanoma must also be excluded in the adult even in tumors without any sign of pigmentation and in patients without history of melanocytic neoplasms, due to the relative frequency of metastatic disease from an occult primary tumor in this kind of lesions. S-100 is the most sensitive diagnostic marker for metastatic melanoma and is negative in most UESL. Nevertheless, focal positivity has occasionally been reported [15], and a confirmation with other melanocytic markers, such as human melanoma black 45 (HMB-45), might be useful in some cases.

In the past, UESL had poor prognosis, with long-term survival rates lower than 37% [8, 21–23]. Even with complete surgical resection of the mass and without evidence of residual disease, more than half of the patients developed local recurrence and/or distant metastases and finally died of disease within two years of the first surgery. After the introduction of multimodal treatment, combining radical surgery, different chemotherapeutic agents, and in some cases, radiotherapy, the patients' outcome has greatly improved and long-term survival rates are reported in the literature around 70% [10, 24, 25]. Nevertheless, there is still no standardized therapy for patients with UESL, and the treatment schemes proposed are based on little case series, mainly on pediatric patients. These schemes were developed starting from those used in other pediatric soft tissue and liver malignancies and include different combinations of vincristine, actinomycin D, doxorubicin, cyclophosphamide, ifosphamide, and cysplatinum [10, 22, 26].

Complete surgical resection with clear margins is currently considered the most important prognostic factor for UESL, both in children and in the adult. Patients with incomplete resection or rupture of tumor mass during surgical procedure have worse prognosis, with no statistically significant improvement in survival rates in those receiving subsequent chemotherapy [4]. On the contrary, the addition of adjuvant chemotherapy to radical surgery significantly improves survival rates.

The major causes of nonresectability of UESL are multifocality inside the liver, portal vein involvement, and distant metastases at the time of diagnosis [27]. Neoadjuvant chemotherapy has shown some success in children in downstaging bulky tumors by making surgical resection possible [10]. Few data are available for the role of liver transplantation in patients with UESL. Some authors recommend liver transplantation in patients with multiple lesions, no evidence of extra hepatic manifestations, and if resection is not possible even after neoadjuvant chemotherapy. However, experience with this treatment strategy is very limited [28].

In summary, UESL in the adult population is very uncommon. From the clinical point of view, these lesions are mainly asymptomatic, they have no specific radiological features, and in most cases, preoperative diagnosis is impossible. Histological diagnosis is important in differentiating this group of rare mesenchymal neoplasms from the more common HCC and hepatoblastoma and may affect the choice of neoadjuvant chemotherapy for bulky unresectable lesions, suggesting the need of needle biopsy in such cases. The ability to achieve radical surgery with clear margins is a fundamental factor that influences the decision to resect the tumor or to refer the patient for neoadjuvant treatment. Liver resection with adjuvant chemotherapy should be offered to all patients with UESL, especially when there are positive margins or tumor rupture during the surgical procedure.
